# Unexplained Hematocrit Increase after Therapeutic Phlebotomy in a Patient with Marked Erythrocytosis

**DOI:** 10.1155/2022/5018388

**Published:** 2022-08-11

**Authors:** Rushad Machhi, Ashley M. Cunningham, Kenneth Hennrick, Karen A. Schaser, Eliot C. Williams, William Nicholas Rose

**Affiliations:** Department of Pathology and Laboratory Medicine, University of Wisconsin Hospital, 600 Highland Ave, D4/250, Madison 53792, WI, USA

## Abstract

We report a patient with hereditary erythrocytosis who underwent a therapeutic phlebotomy and had a post-phlebotomy hematocrit that was higher than the pre-phlebotomy hematocrit. We could not discern a reason for this hematocrit increase after phlebotomy. Instead of performing another phlebotomy, we performed an automated red cell depletion via an apheresis instrument. This procedure is essentially a red cell exchange, but 5% albumin is used as the replacement fluid instead of red blood cells. The patient's hematocrit decreased from 80% to 39% after three consecutive daily red cell depletion procedures. We share our experience to report the unusual finding of a patient's hematocrit that increased with phlebotomy and to raise awareness of the red cell depletion procedure.

## 1. Introduction

Erythrocytosis, or polycythemia, is an elevation of the red blood cell (RBC) mass often recognized through increases in the hemoglobin (Hgb) concentration or hematocrit (Hct). The WHO defines polycythemia as a Hgb concentration or Hct greater than 16.5 g/dL or 49% for males, respectively, or 16.0 g/dL or 48% for females, respectively [1]. The majority of erythrocytosis is incidentally discovered in asymptomatic individuals. However, symptoms and manifestations of erythrocytosis can be quite variable and are typically due to the excess RBC burden resulting in hyperviscosity. Mild symptoms can include fatigue, headaches, and plethora. More severe manifestations, while uncommon, include venous thromboembolism (VTE), myocardial infarction (MI), and cerebrovascular accident (CVA) [2].

Erythrocytosis is often classified into primary and secondary erythrocytosis. Primary erythrocytosis involves alteration in the erythropoietin (EPO) receptor (EpoR) either through an acquired or congenital process [3]. Patients with primary erythrocytosis generally have low levels of EPO and normal oxygen dissociation curves [4]. The most common acquired process is polycythemia vera, which is driven by a somatic mutation in the JAK2 signaling pathway of the EpoR [5]. Less common is hereditary erythrocytosis, which involves a congenital mutation of the EpoR.

Common mutations in hereditary erythrocytosis involve the SHP-1 docking site, which turns off EPO signaling. Patients with hereditary erythrocytosis will have a wild type JAK2 gene [3]. Secondary erythrocytosis, meanwhile, is driven largely by an increase in EPO. Generally, patients will have elevated or inappropriately normal levels of EPO [4]. Common causes of secondary erythrocytosis include chronic hypoxia, EPO producing tumors, and increased androgens. Congenital causes such as 2,3 BPG deficiency, methemoglobinemia, and genetic mutations involving the hypoxia inducing factor (HIF) pathway are less common [3].

The management of secondary erythrocytosis usually requires treating the underlying cause. The management of both primary erythrocytosis and hereditary causes of secondary erythrocytosis usually requires directly decreasing the RBC burden [6]. To that end, the goal of treatment is not only to provide symptomatic relief but also to lower the risk of mortality from the major complications of erythrocytosis. Lowering the hematocrit to a goal of less than 45% has been shown to decrease mortality from major cardiac or thromboembolic events [7].

The mainstay of treatment is therapeutic phlebotomy. Depending on patient size and ability to tolerate an acute decrease in hematocrit, about 500 mL to 1000 mL of whole blood is removed by weighing 500 g to 1000 g, respectively, on a tared blood bag on a scale. In essentially all cases that we have experienced or read about, a therapeutic phlebotomy decreases the hematocrit. If the hematocrit is not lowered enough or if the patient requires frequent intervals of phlebotomy, erythrocytapheresis or red cell depletion (RCD) is an effective and safe alternative. Here, we present the case of a patient with hereditary erythrocytosis whose hematocrit increased after phlebotomy, a finding that appears to be unreported, and then decreased significantly following successive RCDs.

## 2. Case Presentation

A 45-year-old female with a past medical history of hereditary erythrocytosis, juvenile rheumatoid arthritis, and thrombocytopenia presented to the University of Wisconsin Hospital (UWH) with altered mental status, dyspnea, and dysphagia. Chest X-ray showed infiltrates suggestive of pneumonia, and the patient was positive for influenza A. She was admitted and started on a 5-day course of oseltamivir. On admission, her Hct reached a high of 78%, and her Hgb was 22.6 g/dL. The patient had previously been evaluated for hereditary erythrocytosis with negative testing for JAK2 gene exon 14, a normal oxygen dissociation curve, and no significant cardiopulmonary abnormalities. Her EPO levels had been consistently elevated, but the significance of this finding was never clearly found.

After adequate rehydration, a 350 mL therapeutic phlebotomy was performed. However, a post-phlebotomy Hct revealed an increase to 80%. Based on this increase and her small body habitus (patient weight 31.8 kg on admission), the decision to proceed with RCD was made.

RCD was performed via centrifugal apheresis using the Cobe Spectra apheresis system through an internal jugular (IJ) vein hemodialysis line. While RCD is rarely performed, a program for RCD already existed as a program in this machine. The replacement fluid was 5% albumin. No “rinse back” of the whole blood that remained in the machine at the end was performed. The manufacturer, Terumo BCT, was contacted and recommended that the Hct not be lowered by more than 22% per session. However, given her small body habitus and significantly elevated Hct, the decision was made to set the Hct target to 50%. The first RCD lowered her Hct from 80% to 61%. The following day a second RCD with a Hct target of 35% was performed that lowered her Hct to 48%. A third and final RCD was completed the next day with a Hct target of 35% that lowered her Hct from 50% to 39%. About 600 mL of RBCs was removed during each session with an inlet speed of 20 mL/min and whole blood to anticoagulant ratio of 13 : 1. There were no complications or adverse reactions during the procedures. The patient was discharged shortly after completing her RCDs with outpatient follow-up and a plan for regular 500 mL therapeutic phlebotomies.

## 3. Discussion

The key reportable phenomenon was not the use of RCD, which is unusual but reported, but rather the increase in the hematocrit after therapeutic phlebotomy. If this has happened with any regularity, we were not able to find reports of it in our literature search. The main teaching message that we wish to communicate is that if a therapeutic phlebotomy non-intuitively increases your patient's hematocrit, consider this strategy as one option: do not repeat the phlebotomy but do a RCD. Our rationale was that performing another phlebotomy might have increased her hematocrit even higher, and a second increase in hematocrit, however unlikely or unexplainable, seemed like a risk that was unwarranted.

We concede that we do not know why this happened. We can speculate about several possible factors. Perhaps one or more hematocrit results of 78% and 80% were spurious. That is, we speculate that perhaps the hematocrit loses accuracy at the extremes of measurement as the values greatly exceed or recede from the linear or reportable range. After all, the hematocrit is calculated from the MCV and RBC count, so perhaps an extreme RBC count introduces imprecision and/or inaccuracy.

Perhaps the phlebotomy removed relatively more non-RBC fluid and relatively less RBC mass than the proportions that were present in the patient. We can speculate that there may be a rheological explanation that is beyond our expertise. Perhaps when the hematocrit blood becomes that high and the blood becomes that viscous, the physical characteristics of the blood are such that a routine phlebotomy can lead to, for example, relatively more plasma loss and less RBC loss than one would predict. Perhaps one plausible factor is the smaller gauge venous access needle in peripheral access phlebotomy compared to the larger gauge venous access device that was used for the RCDs.

Perhaps the 78% was spuriously low instead of the 80% being spuriously high. For both theories, we have no evidence for or against them, but perhaps the 78% result was relatively diluted by hydration in the patient's body and/or a relatively higher ratio of EDTA anticoagulant to patient sample. Alternatively, the 80% could have been spuriously high from the 2 opposite factors: relative dehydration and/or a relatively lower ratio of anticoagulant to patient sample.

Finally, perhaps the instrument's coefficient of variation was such that 78% and 80% overlap to some degree. This is also beyond our expertise, but we have anecdotal experience with lab test that yields slightly different results when they are repeated nearly simultaneously using the same specimen. Perhaps other factors could explain the increase. We do not claim to have provided an exhaustive list of hypotheses from the fields of laboratory medicine, rheology, or other fields. We stress that our focus is simply to raise awareness of existence of automated red cell depletion and to consider using it if phlebotomy is ineffective for any reason.

The exact etiology of this patient's erythrocytosis is not known with complete clarity. In short, we labeled her with the diagnosis of presumed hereditary erythrocytosis not otherwise specified. Our rationale was that the erythrocytosis appeared to be lifelong and no overt external stimuli appeared to be causing it.

One can suggest additional testing to clarify a specific mutation or labeled syndrome. We admit that we did not go as far as one could in terms of genetic testing for diagnostic or research purposes. Although this patient has not had the full array of genetic testing done to confirm the exact etiology of her hereditary erythrocytosis, based on her chronically elevated EPO levels and normal dissociation curve, this is highly suggestive of a defect in her HIF pathway. Mutations in this pathway commonly arise from the VHL, EGLN2, or EPAS1 genes [4].

As is often the case, we judged that a battery of genetic tests would not be worth the large expense when the result would not be likely to change our management. That is, the probability that any one of these mutations would be found was very low. Also, more importantly, even if one of these mutations had been found, the treatment strategy would almost certainly be the same: to reduce RBC mass via therapeutic phlebotomy (and/or RCD) and, depending on risk stratification, to reduce thrombosis risk (for example, with aspirin). Thus, we judged that we had obtained a sufficient amount of actionable diagnostic information while minimizing the costs.

RCD has been known to be a safe procedure for severe erythrocytosis for many years. In other words, the procedure exists as an option in the most popular therapeutic apheresis machines, but many specialists may not have ever used it and may not be aware of it. Therapeutic apheresis machines and the specialists that use and medically manage them may not be available in all centers. But if the option is available nearby, consider it if your patient has severe erythrocytosis that requires acute treatment, especially if therapeutic phlebotomy is inadequate.

The American Society for Apheresis (ASFA) in their 2019 guidelines reiterated the use of RCD for PV and hereditary erythrocytosis, specifically stating that it can be a useful alternative to phlebotomy in cases where the patient is a high bleeding risk or if red cell reduction is rapidly needed prior to a surgery [6]. RCD treatment has been shown to increase the interval needed between red cell depleting therapies when compared to therapeutic phlebotomy [8].

Furthermore, the complication rate of RCDs has been shown to be as low as 7%, and the complications are usually benign (e.g., mild hypocalcemia from the citrate anticoagulant) [9]. However, IJ line placement usually poses greater risks than RCD itself. Thus, the aggregate risks and benefits of line placement plus RCD should be considered as a bundle. In addition, RCD treatment can cost up to three times more than therapeutic phlebotomy, and this cost is only slightly offset by the increased interval between treatment [10]. Nevertheless, as observed in our case presented here that was expanded from our previous abstract, RCD is a potential option for a patient with severe erythrocytosis that does not respond to therapeutic phlebotomy [11].
